# Global Ophthalmic Health Initiatives in Ebola and Emerging Infectious Disease Outbreaks: Implications for Vision Health Systems, Program Implementation, and Disease Surveillance

**DOI:** 10.1097/IIO.0000000000000443

**Published:** 2022-12-27

**Authors:** Ye Huang, Jalikatu Mustapha, Lloyd Harrison-Williams, Tolulope Fashina, Casey Randleman, Cristos Ifantides, Jessica G. Shantha, Steven Yeh

## Introduction

Globally, the need for eye health services continues to grow and has been deemed a significant public health concern requiring immediate action.^1^ In 2019, an estimated 2.2 billion people worldwide had vision impairment, and at least 1 billion people had preventable or untreated causes of vision loss.^2^ The prevalence of visual disability is predicted to increase with the continued growth of populations and aging. The World Health Organization (WHO) has identified global eye health as a priority and previously launched VISION 2020: The Right to Sight initiative in 1999 to eliminate avoidable blindness globally by 2020.^3^ Although progress was made, not all of the goals for VISION 2020 were met. The WHO then transitioned to the new agenda of implementing universal eye health with global targets set for 2030.^2^,^4^,^5^

Disease outbreaks can have widespread disastrous consequences, drastically increasing the morbidity and mortality within an affected outbreak region with lasting effects. Eye health has been impacted and described in multiple outbreaks, with ophthalmic sequelae occurring in the convalescent phase of infection or during acute disease.^6^,^7^ Thus, in our evaluation of health systems, broad consideration of multiple organ targets, including the eye, can be incorporated as an element of outbreak response, which may include measures for disease detection, clinical phenotyping, understanding of the basis of viral mucosal transmission, and communication of clinical protocols in the care of patients who develop the ophthalmic disease in an emergency response.

Several viral outbreaks are particularly illustrative of the importance of addressing the potential ophthalmic implications that may arise. Following two consecutive outbreaks of yellow fever in Southeastern Brazil, many patients with severe systemic disease were later found to have retinopathy despite a lack of ocular symptoms.^8^ Furthermore, ophthalmic screening of these patients may demonstrate the validity of retinopathy as a prognostic marker. Among infants with congenital Zika virus infection with a small cephalic diameter at birth, fundus abnormalities such as optic disc hypoplasia, and mild pigment mottling with foveal reflex loss were observed.^9^ On the basis of these findings, guidelines are now in place for routine ophthalmic examinations on microcephalic infants with congenital Zika virus infection is recommended to rule out this possibility.^10^

The aftermath of the 2013–2016 Western African Ebola virus disease (EVD) outbreak highlighted many persistent ocular problems that arise following acute disease. Viral persistence, particularly in immune-privileged organs, is a mechanism for organ-specific inflammatory disease during convalescence and may also pose an infectious threat via the potential transmission of Ebola virus (EBOV) from an immune-privileged compartment (eg, sexual transmission from EBOV persistence in the reproductive organs).^11^ One area of ongoing investigation is the pathophysiology of ophthalmic sequelae following EVD infection, specifically, the spectrum of uveitis that may develop in EVD survivors. The absence of a clear correlation between ophthalmic symptoms and a uveitis diagnosis emphasizes a need for regular and prompt ophthalmic screening among EVD survivors, which is a component of the WHO screening guidelines.^12^ Since the Western African EVD outbreak, subsequent outbreaks have occurred, including an outbreak in 2018 in the Democratic Republic of Congo (DRC) that resulted in spillover cases into the neighboring country of Uganda.^13^ While Uganda has been applying lessons learned from prior epidemics with some progress, there remain ongoing weaknesses in areas that include equipment, supplies, number of health care personnel, and infrastructure maintenance.^14^

Furthermore, the ramifications of these ocular complications are oftentimes significant and extend to quality of life and psychosocial wellbeing. Vision impairment has a considerable impact on the functional, social, and psychological capacity of an individual and can subsequently affect the individual’s family and community.^15^ This issue poses a significant burden on a larger scale as well. Inequalities in health typically parallel the socioeconomic status of countries, as seen in global trends of vision impairment.^16^

The high rate of vision loss in West African populations that were studied can be attributed to a number of factors, including knowledge gaps in our understanding of ophthalmic disease before the West African EVD outbreak and weaknesses in vision health systems. The high prevalence of ocular inflammation observed during EVD convalescence emphasizes the need for improved accessibility to the appropriate medical resources in these areas. Moreover, outbreak response affords an opportunity to develop strategies to strengthen vision health systems in preparation for future times of crisis. As we take steps toward strengthening the detection of ophthalmic sequelae following these disease outbreaks, we can also begin to consider the broader implications of global eye health. Herein, we review categories of infectious disease outbreak responses that may have broader implications for vision health systems strengthening.

## Types of Outbreak Response

The process of gathering data related to ophthalmic findings is oftentimes difficult, given that these outbreaks typically occur in resource-limited areas and emergent settings. Obstacles often include a shortage of sufficiently trained health care personnel and the high cost of ophthalmic equipment required for specific assessments. Several strategies can be employed to improve the efficiency of implementing ophthalmic screening programs. These strategies include (1) recruiting nonophthalmologist health workers’ participation in recording findings in outbreak settings using appropriate training methods, (2) rapid response teams to execute ophthalmic screening protocols, and (3) prospective, controlled cohort studies to assess for increasing risk of disease over time.^17^–^19^

## Lessons Learned From EVD

### Ophthalmic Findings in West African EVD Survivors

Among a population of EVD survivors in Libera, 22% were diagnosed with EVD-associated uveitis. Of these patients, 60% showed visual impairment, and 40% were 20/400 or poorer, meeting WHO criteria for blindness.^12^ In the PREVAIL III Study, an NIH-funded longitudinal study of Ebola sequelae in Liberia, a 26.4% prevalence of uveitis among EVD survivors at enrollment was reported.^19^ Interestingly, the prevalence rose to 33.3% in the same cohort 1 year later and was significantly greater than the prevalence of uveitis observed in close contacts of EVD survivors.^19^ These statistics were comparable to a previous series of patients from Sierra Leone, which described a prevalence of 18% to 34% for uveitis found among survivors.^20^,^21^ A cross-sectional study of EVD survivors in Sierra Leone awaiting cataract surgery revealed structural features in affected eyes, including band keratopathy, keratic precipitates, posterior synechiae, uveitic cataracts, and chorioretinal scarring.^22^,^23^

### Ophthalmic Findings in US EVD Survivors

While cases of uveitis arising in EVD survivors outside of West Africa were rare, permanent vision loss was not observed. Several cases documented improvement in vision utilizing a combination of anti-inflammatory medication and experimental antiviral in 1 patient.^22^ One EVD survivor who developed ocular symptoms a month after hospital discharge was found to have findings consistent with a diagnosis of sight-threatening panuveitis. The patient demonstrated immediate improvement after administration of corticosteroids and antiviral medication.^24^ Another patient with EVD who had associated uveitis demonstrated drastic improvement of posterior segment inflammation and visual acuity with initiation of oral prednisone.^25^ These cases highlight the importance of long-term monitoring for uveitis and development of management strategies for these post-EVD inflammatory processes.

### Parallels Between Blindness and Mortality

Disparities in mortality associated with EVD between West Africa and the well-resourced countries have also been described in the literature. The overall case fatality rate in the West African population of Sierra Leone was reported at 74% (range 50% to 90%),^26^ compared to case fatality rate of 19% among patients with EVD who received care in the United States or Europe.^27^ The explanations for these stark differences in mortality reported in West Africa are multifactorial and parallel the vision health outcomes observed in assessments of EVD survivors. Prior evaluation of emergency care capacity in Freetown, Sierra Leone has revealed widespread deficiencies in domains including infrastructure, guidelines for critical care, systems, and training.^28^–^30^ There is also a reported shortage of medical personnel exacerbated by the aftermath of the EVD outbreak, which led to the deaths of 21% of Sierra Leone’s health workforce.^31^ Moreover, the paucity of supportive care and medical countermeasures during the response to the West Africa outbreak differ from the clinical trials and compassionate use protocols utilized in more recent outbreaks in the DRC.^32^–^34^ These considerations emphasize an urgent need for systems strengthening to improve systemic health and vision outcomes.

## Closing the Gap

In the wake of recent EVD outbreaks, initiatives to address the disparities in vision health systems have been implemented. One example is a collaborative effort between ophthalmologists, infectious disease specialists, and eye care nurses to develop a screening eye clinic for EVD survivors in Libera. Resource procurement, clinic and modular design, and infection control were all necessary areas of focus undertaken to achieve this goal.^35^ Other ways to mitigate this issue involve developing specific research questions with in-country partners to address a gap in our current knowledge or understanding. An example of this is the Ebola Virus Persistence in Ocular Tissues and Fluids (EVICT) study that sought to determine EBOV prevalence in survivor eyes requiring cataract surgery, with the goal of using evidence to guide safe and vision-restorative surgery for EVD survivors. Uveitis is estimated to occur in 13% to 34% of EVD survivors, and untreated uveitis may lead to secondary ophthalmic complications, including cataract development. Findings from the EVICT study assisted in the surgical care of patients. Specifically, patients who tested negative for EBOV RNA in ocular fluid specimens promptly received cataract surgery with demonstration of vision-restorative outcomes.^22^ Studies such as these help delineate methodologies that can be applied in these complex situations, including incorporation of community engagement, partnerships, streamlining patient care, and laboratory workflows.^36^ While these projects accomplished delivery of care to smaller cohorts of patients, scalability goals require greater commitments for even broader health systems.

## Vision Health Systems Strengthening—Employing a Staff/Space/Stuff/Systems Approach

In addition to barriers to clinical research that may be faced in well-resourced settings (eg, securing funding, study design development, regulatory approvals), resource-limited countries deal with a plethora of other issues.^36^ This may complicate the process of data collection when studying outbreaks in these areas. Utilization of health system frameworks can achieve systematic examination of eye care disparities in global populations.^37^ One such model that delineates the resources required for health care delivery is the “Four S” (staff, space, stuff, systems) framework (Table 1).^38^ Ophthalmic care for EVD survivors will be used as a case example in Table 1.

**TABLE 1:** Areas of Unmet Need (Staff/Space/Stuff/Systems Approach)

Category	Definition	Importance	Limitations Identified Through Ophthalmic Care for EVD Survivors
Staff	Human capital development/training: ophthalmologists, eye care nurses, technicians	Staff members must be properly trained in imaging techniques and operation of ophthalmic equipment (slit-lamp biomicroscopy, indirect ophthalmoscopy, portable fundus photography, B-scan ultrasound)	In Sierra Leone, previously only 4 ophthalmologists in-country to serve a population of 7 million^36^
Space	Equipment and infrastructure	Appropriately sized clinics, operating theaters, or physical plants to abide by proper precautions for infection control and to allow for efficient screening and imaging, and invasive procedures when needed^38^	Spatial constraints, lack of stable electricity and power Few laboratories able to perform EBOV RT-PCR, preventing same-day RT-PCR analysis, and delaying surgery^36^
Stuff	Supply chain, medications, medical and surgical equipment	Higher volume and increased complexity of patients may raise demand for routine medications, supplies, and equipment	Barriers to patient transportation Shortage of ophthalmic equipment
Systems	Implementation science and operational research methodologies Ophthalmic surveillance in setting of disease outbreak Ophthalmic protocols and policies to respond to epidemics	Systems should be in place and utilized during acute outbreak events to continually reassess demands and ensure that response is adequate	Needs for health governance capacity building in the public sector of low-income countries,^39^ specifically related to the “emergency within the emergency” of immediate eye care needs in EVD survivors^6^

EBOV indicates Ebola virus; EVD, Ebola virus disease; RT-PCR, reverse transcription-polymerase chain reaction.

## Implementation Science

While our prior work in vision health was adequate for programs successfully implemented for screening, viral detection in the eye, and treatment, understanding a broader approach to implementation of programs as a scientific area of study requires rigorous methodology, and efforts remain ongoing. Implementation science studies how to most effectively apply evidence-based practices and assess the success of evidence-based practices application, especially during times of crisis.^40^,^41^ Reach, Effectiveness, Adoption, Implementation, and Maintenance (RE-AIM) and Consolidated Framework for Implementation Research (CFIR) are both frameworks for evaluating the success of program implementation, which warrant preemptive consideration for deployment during public health emergencies and outbreak response.

The RE-AIM framework can be used to translate scientific advances into practice. Components of the RE-AIM framework include dimensions at the individual level: reach (R), effectiveness (E), and maintenance (M), and those at the staff and setting levels: adoption (A), implementation (I), and maintenance (M).^42^ In the ophthalmic setting, RE-AIM has been used to measure implementation of community-based ophthalmic screening programs.^43^

The CFIR is comprised of constructs associated with effective implementation and can be used to explain why an implementation was or was not successful.^44^ CFIR encompasses 5 major domains, which include the intervention, inner setting (ie, structural, political, cultural context through which implementation will proceed), outer setting (ie, economic, political, social context that an organization resides in), individuals involved, and the process by which implementation is achieved. The interaction of these domains with one another influences implementation effectiveness.^45^ Simultaneous application of both frameworks may serve a complementary purpose, with RE-AIM measuring the degree of success and CFIR determining the reason behind the implementation outcome. Frameworks such as these and similar strategies can be employed as we continue to assess implementation strategies used in vision health outbreaks that can be applied to broader public health initiatives. Such strategies could be considered to evaluate ophthalmic response as well.

## Surveillance for Diseases of Ophthalmic Outbreak Consequences

Surveillance is a valuable tool that can be utilized to detect a need for intervention as well as measure the impact of interventions. Decision makers can then use this evidence to inform policies and procedures required for consequence management (eg, vision-threatening eye disease related to EVD, Rift Valley fever, and dengue) and longitudinal follow-up.

*Active surveillance* describes a system in which staff members regularly contact providers or the population to obtain information about health conditions. While this form of surveillance allows for the communication of accurate and timely information, it is also more expensive than passive surveillance.^46^

*Passive surveillance* describes a system in which a health jurisdiction receives reports from hospitals, clinics, or other sources. This is a comparatively less expensive strategy to obtain information from large areas to monitor a community’s health. However, factors such as quality and timeliness are difficult to control because of the reliance on other institutions to provide data.^46^ In addition, while passive surveillance is useful in understanding disease trends, underreporting may occur in certain subpopulations. Integration of active surveillance with passive surveillance may improve early detection of cases and yield a more accurate estimate of disease incidence.^47^

## Conclusion

While over 5 years have passed since the end of the West African EVD outbreak, recent events such as multiple EVD outbreaks within the DRC, the ongoing Uganda epidemic, the COVID-19 pandemic, and the Monkeypox outbreak illustrate the need for an improved understanding of frameworks to approach these global issues. A broader approach for outbreaks with ophthalmic sequelae incorporates vision health systems strengthening that extends beyond addressing the current clinical and logistical challenges. Application of strategies such as implementation science frameworks and surveillance methods may be used to identify, address, and assess program development. Ultimately, if vision health systems are developed in response to the unmet needs and broader care gaps identified in outbreaks, expanded capacity in-country provider capabilities may be better positioned to identify, prevent and treat ophthalmic sequelae associated with emerging infectious disease threats.
